# External Validation of Adjuvant! Online Breast Cancer Prognosis Tool. Prioritising Recommendations for Improvement

**DOI:** 10.1371/journal.pone.0027446

**Published:** 2011-11-08

**Authors:** David Hajage, Yann de Rycke, Marc Bollet, Alexia Savignoni, Martial Caly, Jean-Yves Pierga, Hugo M. Horlings, Marc J. Van de Vijver, Anne Vincent-Salomon, Brigitte Sigal-Zafrani, Claire Senechal, Bernard Asselain, Xavier Sastre, Fabien Reyal

**Affiliations:** 1 Department of Biostatistics, Institut Curie, Paris, France; 2 Department of Radiotherapy, Institut Curie, Paris, France; 3 Department of Tumour Biology, Institut Curie, Paris, France; 4 Department of Medical Oncology, Institut Curie, Paris, France; 5 Department of Pathology, Academic Medical Center, Amsterdam, Netherlands; 6 Department of Surgery, Institut Curie, Paris, France; Health Canada, Canada

## Abstract

**Background:**

Adjuvant! Online is a web-based application designed to provide 10 years survival probability of patients with breast cancer. Several predictors have not been assessed in the original Adjuvant! Online study. We provide the validation of Adjuvant! Online algorithm on two breast cancer datasets, and we determined whether the accuracy of Adjuvant! Online is improved with other well-known prognostic factors.

**Patients and Methods:**

The French data set is composed of 456 women with early breast cancer. The Dutch data set is composed of 295 women less than 52 years of age. Agreement between observation and Adjuvant! Online prediction was checked, and logistic models were performed to estimate the prognostic information added by risk factors to Adjuvant! Online prediction.

**Results:**

Adjuvant! Online prediction was overall well-calibrated in the French data set but failed in some subgroups of such high grade and *HER2* positive patients. *HER2* status, Mitotic Index and Ki67 added significant information to Adjuvant! Online prediction. In the Dutch data set, the overall 10-year survival was overestimated by Adjuvant! Online, particularly in patients less than 40 years old.

**Conclusion:**

Adjuvant! Online needs to be updated to adjust overoptimistic results in young and high grade patients, and should consider new predictors such as Ki67, HER2 and Mitotic Index.

## Introduction

Breast cancer prognosis determination relies upon pathological features able to classify breast cancer in subgroups of similar behavior. Tumour size, axillary lymph node involvement, grade as defined by Elston & Ellis, Estrogen Receptor (ER) status, Progesterone Receptor (PR) status and *HER2* status are routine determinants of the breast cancer prognosis. To improve the ability to accurately predict the prognosis of breast cancer patients and the likely benefit of adjuvant systemic therapy, combinations of several clinicopathological prognostic factors have been tailored for clinical decision making. Nottingham Prognosis Index (NPI) is based on the Tumour Size, Tumour Grade and Lymph Node Involvement [NPI = Grade+Node+0.2*Size] [1], [2], [3]. More recently Ravdin *et al* [4] built a web based application able to provide 10 years survival and relapse probability of an individual patient (www.adjuvantonline.com). This index is derived from women 35 to 59 years old at diagnosis and treated between 1988 and 1992 of the US SEER (Surveillance, Epidemiology, End-Results data and estimates) registry which includes around 10% of all US breast cancer patients. The calculation process is based on actuarial analysis using age at diagnosis, comorbidity factors, ER, tumour size, tumour grade and lymph node status as input. The program gives the estimated prognosis, but also the expected benefit of several therapeutic options in a comprehensive format, adapted for decision making. Estimates of the efficacy of adjuvant hormonal therapy and chemotherapy are based mainly on the proportional risk reduction reported by Early Breast Cancer Trialists Collaborative Group meta-analyses [5], [6], [7].

Although Adjuvant! Online is increasingly being used by physicians[8], few validation studies from different countries with very different patient and tumour patterns have been published [9], [10], [11], [12]. Some limitations have been underlined in these studies, including 1) Adjuvant! Online's prediction were on the whole overoptimistic on a UK population; 2) Young age seems to be a category with constant overestimated probability; 3) Relapse free survival estimation, based on extrapolation, seems to be unreliable.

Finally, several predictors such as *HER2* over expression status, proliferation markers or gene expression signatures [13] have not been assessed in the original Adjuvant! Online study. Although these prognostic factors are more and more introduced into clinical practice, no validation study has evaluated the performance of Adjuvant! Online algorithm among subgroups defined with these variables.

The aim of this study was to validate Adjuvant! Online algorithm on two breast cancer datasets collected from two large European comprehensive cancer centres, and to determine whether the accuracy of Adjuvant! Online could be improved by the use of several well-known prognostic factors not used in current calculations for the prediction of 10- year overall survival.

## Materials and Methods

### Ethics Statement

The registration of patients of the Institut Curie in this cohort received a favorable agreement of the french National Committee on Computers and Liberties (CNIL, Commission nationale de l'informatique et des libertés). Patients gave informed written consent prior to be registered in the cohort. The study was approved by the breast cancer study group of the Institut Curie. Dutch study was approved by the medical-ethics committee of the Netherlands Cancer Institute.

### Patients

#### French data set

The French population is an original data set composed of 456 women treated at the Institut Curie between 1995 and 1996. This data set was firstly collated to validate the Ki67 rate and other factors prognostic value in a larger cohort of early-stage breast cancer patients. Inclusion criteria were early breast cancer, without lymph node involvement, initially treated by conservative surgery and radiotherapy. None of these patients received neoadjuvant treatment (chemotherapy, hormonotherapy or radiotherapy). The clinical data were extracted from medical records of the Institut Curie prospective breast cancer database. Histological features (histological type, histological grade as defined by Elston & Ellis, Mitotic Index, Ki67 staining, ER status, PR status, *HER2* over-expression status) were re-assessed for each sample by pathologists of the Institut Curie. Mitotic Index (MI) was defined by the number of mitoses observed in 10 successive high power fields using a microscope with a 40x/0.7 objectives and a 10x ocular. Mitotic Index was assessed on histological sections stained by Hematein, Eosin and Saffron. Van Diest and al's criteria [14] were used to define mitotic figures. Ki67 (Clone MIB1,Dako A/S, Glostrup, Denmark) immunostaining was performed. Semiquantitative assessment was performed by estimating at X200 magnification, the percentage of positive neoplastic nuclei within the area of highest positivity chosen after scanning the entire tumour surface at low power (x10 objective). All nuclei with homogeneous staining, even with a light staining or only a nucleolar staining, were interpreted as positive. ER (clone 6F11, Novocastra, 1/200), PR (clone 1A6, Novocastra, 1/200) immunostaining were performed as previously described. Cases were considered positive for ER and PR according to standardised guidelines using ≥10% of positive nuclei per carcinomatous cells. *HER2* overexpression status was determined according to American Society of Clinical Oncology (ASCO) guidelines [15].

#### Dutch data set [16]

Tumors from a series of 295 consecutive women with breast cancer were selected from the fresh-frozen–tissue bank of the Netherlands Cancer Institute according to the following criteria: the tumor was primary invasive breast carcinoma that was less than 5 cm in diameter at pathological examination (pT1 or pT2); without involvement of the apical axillary lymph nodes; the age at diagnosis was 52 years or younger; the calendar year of diagnosis was between 1984 and 1995; and there was no previous history of cancer, except non-melanoma skin cancer. In the original study, this population was used to validate the prognostic value of a 70-gene expression profile. All patients had been treated by modified radical mastectomy or breast-conserving surgery, including dissection of the axillary lymph nodes, followed by radiotherapy if indicated. Follow-up information was extracted from the medical registry of the Netherlands Cancer Institute. Formalin-fixed, paraffin embedded tumour tissue was used to evaluate the following: tumour type, histological grade, Mitotic Index, ER status, *HER2* status.

In both data sets, Adjuvant! Online version 8.0 was used to compute 10-years survival probability.

### Statistics

All computations were done separately on French and Dutch data sets. Analysis included two stages. First, we checked the agreement between observed and Adjuvant! Online predicted 10-years survival, and secondly, to what extent each prognostic factor adds some information to Adjuvant! Online prediction.

#### Agreement between observed 10-years survival and Adjuvant! Online predicted 10-years survival

Average Adjuvant! Online predicted 10-years survival and observed 10-years survival were computed globally and for several subgroups of patients defined from major prognostic factors in breast cancer. Calibration between observed and mean predicted outcome was evaluated using the Cox method [17]. This method tests the agreement between a sequence of binary responses Y_i_ (i.e. 10-year observed survival yes = 1 or no = 0, for each patient i) and a set of corresponding probabilities p_i_ (ie Adjuvant! Online 10-year predicted survival probability), by fitting a simple logistic model without intercept: log(P(Y_i_ = 1)/P(Y_i_ = 0)) = α+β log(p_i_/(1−p_i_)), and testing the null hypothesis that intercept α is equal to 0 and β is equal to 1, corresponding to a well calibrated model.

#### Performance of Adjuvant! Online and other predictors

If a lack of agreement between observation and prediction is detected in some specific subgroups, some variables were tested as to whether they would add prognostic information to Adjuvant! Online. Variables already used in Adjuvant! Online algorithm and variables not used in Adjuvant! Online algorithm were evaluated in two separated analyses. To perform these two analyses, we used a method derived from Bleeker *et al* [18] to test the contribution of each factor to Adjuvant! Online prediction: a logistic regression model was performed including Adjuvant! Online survival prediction as an offset variable, i.e. with a parameter estimate constrained to 1. This analysis allows estimating the regression coefficient β of a variable X_i_ taking into account AdjvuvantOnline *a priori* known component by including p_i_ in the linear predictor during fitting: log(P(Y_i_ = 1)/P(Y_i_ = 0)) = α+βX_i_+log(p_i_/(1−p_i_)). Since the parameter of the offset variable is equal to 1, Adjuvant! Online prognostic value is not re-estimated in the model.

Variables already used by Adjuvant! Online for 10-year survival prediction (i.e. age at diagnosis, ER status, tumour size, tumour grade, lymph node status and treatment type) were included together in a multivariate model with Adjuvant! Online prediction offset. In this model, a significant coefficient means that the association of a predictor with the outcome in the validation population differs from the original population [18].

Other variables not used in Adjuvant! Online algorithm (histologic type, HER2 status, mastectomy, Mitotic Index, KI67 and gene-expression signature if available) were included separately in an univariate model only with Adjuvant! Online prediction offset. In this analysis, a significant coefficient means that a predictor adds some information to Adjuvant! Online original model.

The gain in predictive inaccuracy of models including variables not used in Adjuvant! Online algorithm, as compared with model with Adjuvant! Online prediction alone, was investigated using the method of Schemper [19]. Reyal and colleagues have recently used this approach in the context of gene signatures [20]. Predictive inaccuracy is calculated as the average of the absolute difference between observed outcomes and model predictions. Explained variations were also computed and represent a measure equivalent to *R*2 in linear regression. Standard errors were obtained by bootstrapping 200 resamples. Areas under the curve (AUC) were also computed.

Significance was defined as p<0.05 (two-sided). Statistical analyses were performed using R 2.12.1 software (http://www.R-project.org).

## Results

### Baseline characteristics

#### French data set

10-years survival status was unknown for 21 patients. Four hundred and thirty five (435) out of 456 patients were included in the analysis. Patients included in the analysis were compared to those not included; no statistically significant difference was observed between this two groups.

Patients' clinico-pathological characteristics are shown in **Table 1**. Mean age was 55 years old (±8.4) at time of diagnosis. The majority of patients had an invasive ductal carcinoma (78%), a T1 tumor (76%), Elston Ellis grade 1 (36%) or grade 2 tumours (42%) and received no adjuvant systemic treatment nor hormonal therapy (87%).

**Table 1:** Baseline characteristics, French and Dutch population.

		French data set	Dutch data set	
		*N (%)*	*N (%)*	*P*
All patients		435 (100)	247 (100)	
Age (years)		54.6 (8.42) a	43.7 (5.5) a	<0.01
	<40	16 (3.7)	57 (23.1)	
	≥40	419 (96.3)	190 (76.9)	
Oestrogen-receptor status	Positive	367 (84.4)	176 (71.3)	<0.01
	Negative	68 (15.6)	71 (28.7)	
Histology	Ductal carcinoma	340 (78.2)	233 (94.3)	<0.01
	Lobular carcinoma	63 (14.4)	10 (4.0)	
	Other	32 (7.4)	4 (1.6)	
Tumour size (mm)	≤20	328 (75.4)	124 (50.2)	<0.01
	>20	107 (24.6)	123 (49.8)	
Grade	1	156 (35.9)	44 (17.8)	<0.01
	2	182 (41.8)	81 (32.8)	
	3	97 (22.3)	122 (49.4)	
N	Positive	0 (0)	120 (48.6)	<0.01
	Negative	435 (100)	127 (51.4)	
HER2	Positive	23 (5.3)	47 (20.6)	<0.01
	Negative	412 (94.7)	181 (79.4)	
Treatment	None	377 (86.7)	139 (56.3)	<0.01
	Chemotherapy	28 (6.4)	79 (32.0)	
	Hormonotherapy	30 (6.9)	14 (5.7)	
	Both	0 (0)	15 (6.1)	
Mastectomy	No	435 (100)	140 (56.7)	<0.01
	Yes	0 (0)	107 (43.3)	
Mitotic index	1	298 (68.5)	95 (38.5)	<0.01
	2	53 (12.2)	38 (15.4)	
	3	84 (19.3)	114 (46.2)	
KI67	≤20	262 (49.9)	-	
	>20	173 (50.1)	-	
Genomic signature	Negative	-	155 (62.8)	
	Positive	-	92 (37.2)	

a mean (sd).

#### Dutch data set

10-year survival status was unknown for 48 patients. 247 out of 295 patients were included in the analysis. Patients included in the analysis were more likely to have negative ER and grade III tumors.

Dutch patients were younger than the French (44±5.5 years), and tumors showed more aggressive features: 50% had T2 tumors, and 49% had grade III tumors. More than 35% of patients had undergone chemotherapy.

### Calibration and models

#### French data set

**Figure 1:**
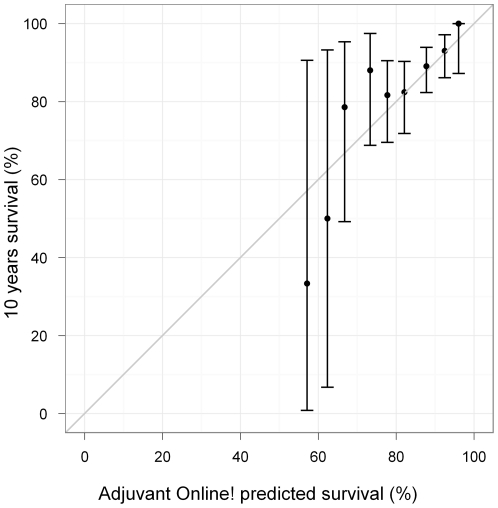
Mean predicted versus observed survival. French population. The data were divided into 5% intervals for the predicted values. Observed percentages were calculated for each interval subset and were plotted against the average predicted values. The grey thin line of slope = 1 and intercept = 0 corresponds to a perfect agreement between observed and predicted values.

The overall 10-year survival of the French population was very close to the Adjuvant! Online predicted survival (87% vs. 85%). **Figure 1** shows the observed versus the average predicted outcomes in the overall French population. Adjuvant! Online was globally well calibrated. Observed and predicted outcomes did not differ regardless patients' predicted prognoses (p = 0.35). However, a subgroup analysis highlighted some weaknesses in the performance of Adjuvant! Online prediction in, for instance, high grade, Ki67<20 and chemotherapy treated tumors subgroups (**Table 2**). 10-year survival in patients younger than 40 years old was overestimated by Adjuvant! Online, but the difference was not significant (75% vs. 90%, p = 0.22).

**Table 2:** Adjuvant! Predicted versus observed 10 years survival.

			Agreement evaluation			
			*Predicted survival* a	*Observed survival* a	*Predicted - Observed*	*P*
	*All patients*		85.1	87.4	−2.3	0.35
**Adjuvant! Online predictors**	*Age (years)*	<40	89.5	75.0	14.5	0.22
		≥40	84.9	87.8	−2.9	0.21
	*Estrogen receptor status*	Positive	86.3	89.6	−3.3	0.14
		Negative	78.5	75.0	3.5	0.18
	*Tumour size (mm)*	≤20	87.9	89.6	−1.7	0.60
		>20	76.5	80.4	−3.9	0.58
	*Grade*	1	89.9	94.2	−4.3	0.14
		2	84.7	89.6	−4.9	0.09
		3	77.9	72.2	5.7	0.02
	*Treatment*	None	85.4	89.9	−4.5	0.02
		Chemotherapy	81.4	71.4	10	<0.01
		Hormonotherapy	84.4	70.0	14.4	0.04
		Both				
**Other predictors**	*Histology*	Ductal carcinoma	84.7	87.1	−2.4	0.37
		Lobular carcinoma	86.3	87.3	−1	0.85
		Other	86.2	90.6	−4.4	0.70
	*HER2*	Positive	82.7	69.6	13.1	0.07
		Negative	85.2	88.4	−3.2	0.16
	*Mitotic index*	1	84.4	92.6	−8.2	0.01
		2	83.9	79.2	4.7	0.30
		3	77.5	73.8	3.7	0.19
	*KI67*	<20	87.4	92.7	−5.3	0.01
		≥20	81.5	79.2	2.3	0.06

French population.

a Percentage.

Variables significantly associated with 10-year survival were Elston Ellis grade, *HER2* over expression status, Mitotic Index and Ki67 index (data not shown). Taking into account Adjuvant! Online information as an offset in the logistic regression model, grade became non significant (**Table 3**). *HER2* status, Mitotic Index, Ki67 and treatment type were strongly associated with 10-year survival, even considering this Adjuvant! Online *a priori* information.

**Table 3:** Odds ratio taking into accout Adjuvant Online! a priori information.

			Predictor evaluation	
			*OR* a *[CI 95%]*	*P*
**Adjuvant! Online predictors** b	*Age (years)*	<40	1	0.12
		≥40	3.26 [ 0.82; 13.05 ]	
	*Estrogen receptor status*	Positive	1	0.69
		Negative	0.85 [ 0.37; 1.92 ]	
	*Tumour size (mm)*	≤20	1	0.33
		>20	1.37 [ 0.72; 2.63 ]	
	*Grade*	1	1	0.18
		2	0.91 [ 0.39; 2.12 ]	
		3	0.48 [ 0.19; 1.21 ]	
	*Treatment*	None	1	0.02
		Chemotherapy	0.66 [ 0.22; 1.98 ]	
		Hormonotherapy	0.27 [ 0.11; 0.66 ]	
		Both		
**Other predictors** c	*Histology*	Ductal carcinoma	1	0.86
		Lobular carcinoma	0.89 [ 0.39; 2.02 ]	
		Other	1.31 [ 0.37; 4.7 ]	
	*HER2*	Positive	1	0.04
		Negative	2.98 [ 1.12; 7.95 ]	
	*Mitotic index*	1	1	0.02
		2	0.39 [ 0.17; 0.88 ]	
		3	0.44 [ 0.23; 0.86 ]	
	*KI67*	<20	1	0.01
		≥20	0.46 [ 0.25; 0.84 ]	

French population.

a associated odds ratio taking into account Adjuvant! prediction.

b multivariate estimation.

c univariate estimation.

#### Dutch data set

**Figure 2:**
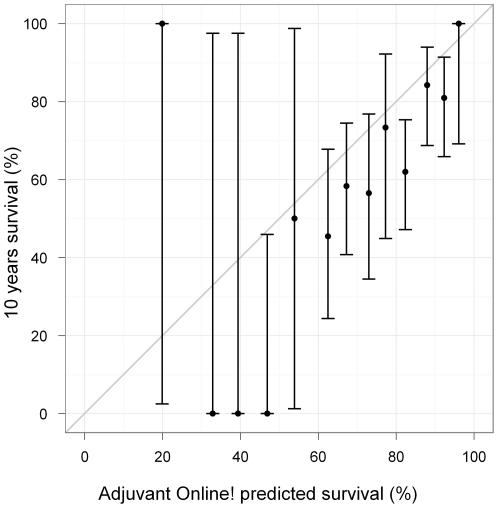
Mean predicted versus observed survival. Dutch population. The data were divided into 5% intervals for the predicted values. Observed percentages were calculated for each interval subset and were plotted against the average predicted values. The thin line of slope = 1 and intercept = 0 corresponds to a perfect agreement between observed and predicted values.

The overall 10-year survival was overestimated by Adjuvant! Online and observed and predicted outcome differed significantly (66% vs. 79% p = 0.00001) (**Table 4** **,** **Figure 2**). This difference was mainly due to age: the larger difference between prediction and observation was observed for the subgroup of patients younger than 40 years old at diagnosis (75.7% vs. 45.6%, p<0.01). Age at diagnosis, Elston Ellis grade, *HER2* status, Mitotic Index, and 70-genes signature were significantly associated with 10-years survival (data not shown). Only age, Mitotic Index and 70-genes signature remained significant when analyzed with Adjuvant! Online information as offset in the logistic regression model (**Table 5**).

**Table 4:** Adjuvant! Predicted versus observed 10 years survival.

			Agreement evaluation			
			*Predicted survival* a	*Observed survival* a	*Predicted - Observed*	*P*
	All patients		78.6	66.4	12.2	<0.01
**Adjuvant! Online predictors**	Age (years)	<40	75.7	45.6	30.1	<0.01
		≥40	79.5	72.6	6.9	0.06
	Estrogen receptor status	Positive	82.8	75.0	7.8	0.01
		Negative	68.3	45.1	23.2	<0.01
	Tumour size (mm)	≤20	86.5	74.2	12.3	<0.01
		>20	70.6	58.5	12.1	0.01
	N	No	81.2	64.6	16.6	<0.01
		Yes	75.9	68.3	7.6	0.13
	Grade	1	92.1	95.5	−3.4	0.59
		2	84.3	76.5	7.8	0.02
		3	70.0	49.2	20.8	<0.01
	Treatment	None	78.3	64.0	14.3	<0.01
		Chemotherapy	79.7	68.4	11.3	0.05
		Hormonotherapy	71.5	64.3	7.2	0.41
		Both	82.4	80.0	2.4	0.94
**Other predictors**	Histology	Ductal carcinoma	78.5	66.1	12.4	<0.01
		Lobular carcinoma	78.5	70.0	8.5	0.75
		Other	85.2	75.0	10.2	0.31
	Mastectomy	No	81.1	70.7	10.4	<0.01
		Yes	75.4	60.7	14.7	<0.01
	HER2	Positive	72.3	51.1	21.2	<0.01
		Negative	79.7	69.1	10.6	<0.01
	Mitotic index	1	88.1	87.4	0.7	0.89
		2	82.6	73.7	8.9	0.06
		3	69.4	46.5	22.9	<0.01
	Signature	Negative	75.3	51.0	24.3	<0.01
		Positive	84.3	92.4	−8.1	0.04

Dutch population.

a Percentage.

**Table 5:** Odds ratio taking into accout Adjuvant Online! a priori information.

			Predictor evaluation	
			*OR* a *[CI 95%]*	*P*
**Adjuvant! Online predictors** b	*Age (years)*	<40	1	0.01
		≥40	2.61 [ 1.31; 5.19 ]	
	*Estrogen receptor status*	Positive	1	0.60
		Negative	0.83 [ 0.42; 1.66 ]	
	*Tumour size (mm)*	≤20	1	0.09
		>20	1.73 [ 0.92; 3.25 ]	
	*N*	No	1	0.09
		Yes	0.44 [ 0.17; 1.12 ]	
	*Grade*	1	1	0.08
		2	0.33 [ 0.07; 1.50 ]	
		3	0.23 [ 0.05; 1.05 ]	
	*Treatment*	None	1	0.55
		Chemotherapy	0.48 [ 0.18; 1.29 ]	
		Hormonotherapy	0.68 [ 0.17; 2.75 ]	
		Both	0.64 [ 0.13; 3.16 ]	
**Other predictors** c	*Histology*	Ductal carcinoma	1	0.96
		Lobular carcinoma	1.21 [ 0.28; 5.31 ]	
		Other	0.91 [ 0.07; 11.76 ]	
	*Mastectomy*	No	1	0.61
		Yes	0.86 [ 0.49; 1.52 ]	
	*HER2*	Positive	1	0.26
		Negative	1.50 [ 0.75; 3.00 ]	
	*Mitotic index*	1	1	0.02
		2	0.62 [ 0.24; 1.63 ]	
		3	0.38 [ 0.18; 0.79 ]	
	*Signature*	Negative	1	<0.01
		Positive	8.21 [ 3.41; 19.72 ]	

Dutch population.

a associated odds ratio taking into account Adjuvant! prediction.

b multivariate estimation.

c univariate estimation.

### Predictive accuracy

The gain in predictive accuracy from adding each variable not used in Adjuvant! Online algorithm is presented in **Table 6** and **Table 7**. In the French data set, the largest decrease in predictive inaccuracy is seen with Mitotic Index, following with Ki67 and *HER2* over expression (3.3%, 2.4% and 1.2% in term of explained variation, respectively). Values for explained variation are higher in the Dutch data set, particularly for 70-genes signature (13.1%) and Mitotic Index (6.7%). Results were similar in term of gain of area under the curve.

**Table 6:** Predictive inaccuracy, explained variation and area under curve for 10-years survival in the French dataset.

	Predictive inaccuracy	Explained variation *(%)*	AUC
Model without predictors	0.212±0.021	−	0.652 [0.575;0.728]
Model with histological type	0.212±0.022	0.0±0.5	0.655 [0.579;0.731]
Model with HER2	0.210±0.021	1.2±1.7	0.678 [0.606;0.750]
Model with mitotic index	0.205±0.021	3.3±2.1	0.702 [0.633;0.771]
Model with KI67	0.207±0.020	2.4±1.7	0.692 [0.625;0.760]

All models are estimated taking into account Adjuvant! Prediction as an offset.

**Table 7:** Predictive inaccuracy, explained variation and area under curve for 10-years survival in the Dutch dataset.

	Predictive inaccuracy	Explained variation *(%)*	AUC
Model without predictors	0.393±0.017	−	0.701 [0.634;0.769]
Model with histological type	0.393±0.017	0.0±0.5	0.701 [0.634;0.769]
Model with surgery	0.392±0.017	0.3±0.9	0.707 [0.641;0.774]
Model with HER2	0.396±0.019	−0.6±2.1	0.700 [0.631;0.769]
Model with mitotic index	0.367±0.023	6.7±3.2	0.738 [0.674;0.802]
Model with genomic signature	0.342±0.019	13.1±3.6	0.775 [0.718;0.832]

All models are estimated taking into account Adjuvant! Prediction as an offset. Data are presented as the mean ± standard error.

## Discussion

Using two independent and very different data sets, this study focused on evaluation of the 10-year survival prediction performance of Adjuvant! Online. The French dataset was composed of patients with an early stage breast carcinoma with no axillary lymph node involvement. Few patients received an adjuvant systemic treatment. The Dutch Dataset was previously published as the validation study of the 70-genes molecular signatures. It was composed of patients younger than 52 years old and a significant over-representation of patients with aggressive features (young age, high grade, HER2 positive, ER negative) was identified when compared to the French Dataset. We showed that the calibration of Adjuvant! Online was overall satisfactory in the French data set, but failed in some subgroups of patients, particularly among those with the most aggressive prognostic factors (young age, high grade and *HER2* positive patients). In the Dutch data set, Adjuvant! Online prediction was highly overoptimistic.

The 10-year survival estimation error in the subgroup of the youngest patients was already highlighted in three other large calibration studies [9], [10], [11]. In the French data set, 10-year survival of patients younger than 40 years old was overestimated by Adjuvant! Online, and the difference was significant. The difference was more obvious in the Dutch validation set that included only patients younger than 52 years old. 23% of them were younger than 40 years old. Consequently, outcome prediction in this subgroup of patients should be interpreted carefully in the Dutch population: the weak calibration of Adjuvant! Online was identified in almost all subgroups and could be explained by the over-representation of young patients in the overall population. Our discussion will therefore mainly focus on results based on the French data set, since Adjuvant! Online calibration was overall correct.

**Figure 3:**
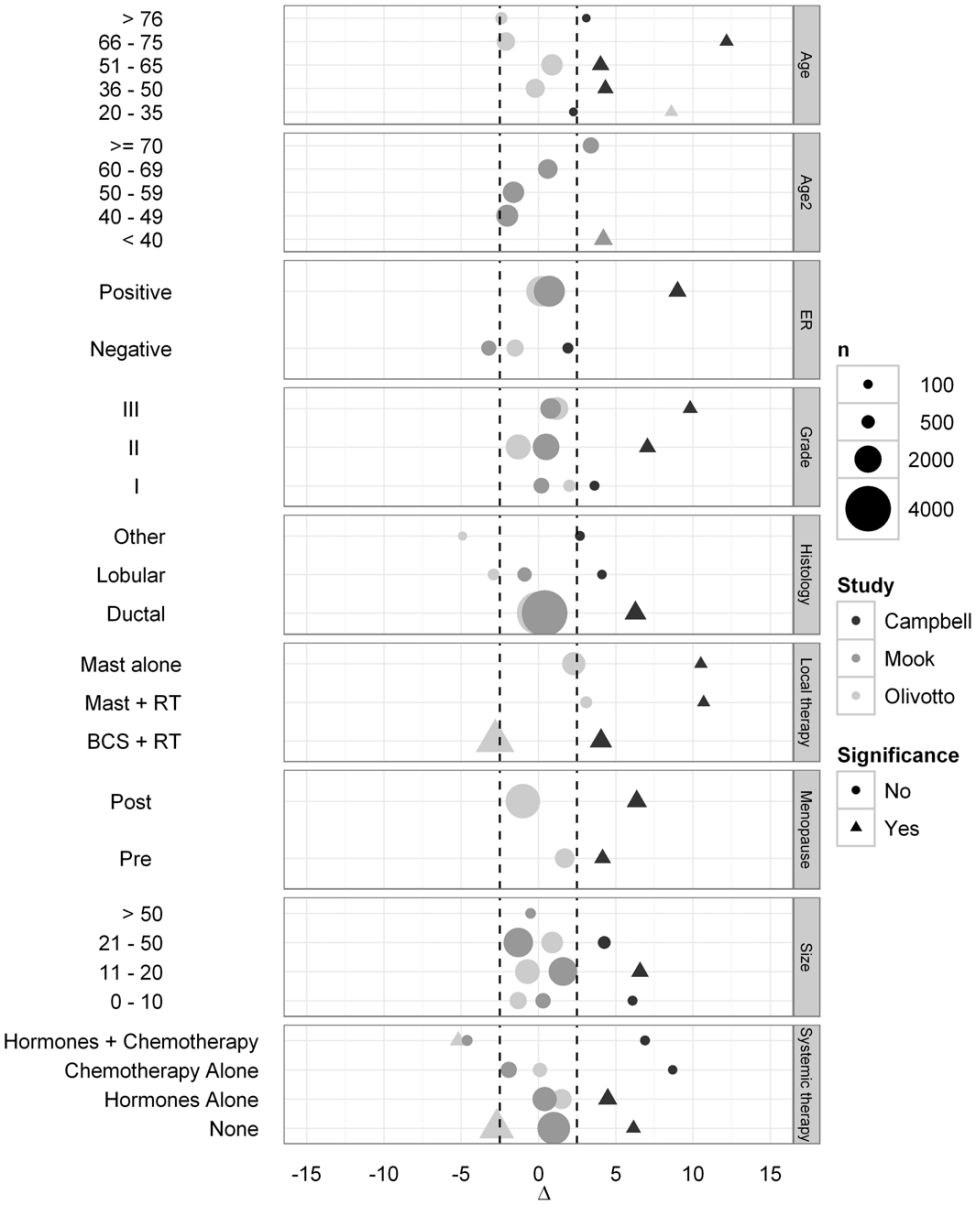
Difference (Δ) between observed outcome and Adjuvant! Online prediction in three other major confirmation studies [9], [10], [11].

Adjuvant! Online survival estimation was restricted to women in the SEER registry from 35 to 59 years of age at diagnosis [4]. Prediction for patients younger that 35 or older than 59 relies mostly on approximations. Although these subgroups have small sample size, **Figure 3** showed that predictions were overoptimistic in two validation study [9], [11]. Some adjustments can be done using the prognostic factor impact calculator integrated in the Adjuvant! Online tool [4], [9], but the manual agreement obtained between observation and prediction relies on operator judgement. In our opinion, this process does not meet the requirement of a prognostic index in individual-based clinical practices because of the lack of reproducibility between estimation. Specific model should be designed in these subgroups, and Adjuvant! Online predictions should only be computed and used for 35 to 59 years old patients.

In the French data set, survival of patients with grade 3 tumors seemed to be overestimated by Adjuvant! Online. Campbell *et al* [9] found a similar result and even concluded that Adjuvant! Online predictions were overoptimistic in almost all subgroups analysis performed. In our study, the prognostic value of grade as defined by Elston Ellis became non significant when taking into account Adjuvant! Online *a priori* information. Thus, the global effect of grade on 10-year survival seems to be captured by Adjuvant! Online, but is underestimated in the subgroup of grade 3 tumors.

To our knowledge, our study is the first to evaluate the influence of *HER2* status, Ki67 and Mitotic Index on the performance of Adjuvant! Online. In the French data set, Adjuvant! Online was over optimistic in *HER2* positive, Mitotic Index>1 or Ki67>20 patients. All three factors brought some statistically significant prognostic information in addition to Adjuvant! Online. These results are consistent with a recent finding from Lende *et al* [21] showing that Mitotic Index is superior to Adjuvant! Online guidelines in prognosticating patients with lymph-node negative breast cancer younger than age 55 years. However, predictive accuracy and AUC improvement when taking into account these variables remained small. This was expected since the overall calibration of Adjuvant! Online was correct. But the interest of these variables should be considered in term of individual prediction: the underestimation of mortality in the small subgroups of *HER2*+ patients do not change a lot the global accuracy of the model, but can have serious individual consequences, since these estimations may be utilized by clinicians to change their therapeutic attitude [8], [22], [23], [24], besides other factors also taken into consideration [24], [25]. Financial implications should of course be discussed, and the minor global improvement of Adjuvant! Online performance could be considered too weak in term of cost-effectiveness, given the scarcity of health care resources. On the other hand, Adjuvant! Online is not necessarily the most cost-effective tool currently available [26]. Moreover, *HER2* [23] and Mitotic Index are already used in clinical practices, and the use of Ki67 measure should increase, since its prognostic value has been confirmed in several studies, including univariate and multivariate models [27]. Absence of HER2 overexpression. and low Ki67 may also identify patients who obtain minimal benefit from adjuvant chemotherapy [28].

Mitotic Index and Ki-67 are both proliferative biomarkers that were correlated in early breast cancers treated with neoadjuvant therapy [29]. Despite a slight advantage of Mitotic Index over Ki67 in terms of improvement of the predictive inaccuracy of Adjuvant! Online, this study was not enough powered to assert that one factor is statistically better than one other, and if both add independent prognostic information in a multivariate model.

Despite of the fact that results from Dutch population must be interpreted with caution, it seems important to highlight that genomic information could improve prediction accuracy in breast cancer. Besides Amsterdam 70-gene prognostic signature [16], several authors [30], [31], [32] have validated the independent prognostic information brought by molecular characteristics of the tumours, and emphasized the complementary nature of these tools with more classical clinico-pathological features.

This study is not without limitations. First of all, Adjuvant! Online was developed in the United States. As in Campbell *et al* [9] British study conclusion, lack of agreement between observation and Adjuvant! Online prediction could come from a poor external validity of Adjuvant! Online in French or Dutch population, and could confirm the need for a state-specific prediction model development. Using an American population, the gap between observation and prediction results could be potentially smaller. However, the prognostic value of *HER2* status, Ki67 and Mitotic Index are well documented in many publications [33], [34], and the growing use of these validated independent prognostic factors in clinical practices should lead to consider them as good candidate markers to improve the accuracy of actual model.

Another weakness of this study is the small sample size of the two validation sets, compared to other validation studies. When the sample size is large, a classical approach is to group subjects into sets with nearly constant predicted probability, and to compare observed proportion with prediction in each set. However, this method is not adapted for small sample size, since it leads to very small sets. That is why we used the Cox approach, specifically developed to test the calibration between observation and prediction with small sample size [17].

The use of a Kaplan-Meïer estimations and proportional hazard models would have been more adapted to describe survival data, like in other validation studies [9], [10], [11]. Nevertheless, the use of a logistic model has a specific advantage: by introducing Adjuvant! Online prediction as an offset into the model, this method allows to test directly if a factor adds or not some prognostic information to the current Adjuvant! Online prediction. Since Adjuvant! Online tool only provides 10-year survival probabilities and does not provide any coefficients of the underlying model used [4], [35], [36], it was not possible to introduce Adjuvant! Online prediction in a survival model.

In conclusion, over optimistic results of Adjuvant! Online in poor prognostic subgroups are problematic, since some therapeutic decisions are based on Adjuvant! Online results. Adjuvant! Online could therefore be updated to adjust biased predictions in young and high grade subgroups of patients, and to consider several other candidate markers, especially Ki67, *HER2,* and Mitotic Index, known as independent risk factors in breast cancer. These recommendations are based on the analyses of two relatively small data sets, and should be validated in an independent population.
